# A blueprint for improving undergraduate education in intensive care medicine

**DOI:** 10.1186/s13054-016-1383-5

**Published:** 2016-07-27

**Authors:** Enda O’Connor, Ignacio Martin-Loeches

**Affiliations:** Department of Intensive Care Medicine, St James’s University Hospital, Trinity Centre for Health Sciences, James’s Street, Dublin 8, Ireland

According to the World Federation of Medical Schools, medical students should have “sufficient knowledge and clinical and professional skills to assume appropriate responsibility after graduation” [1]. Some medical schools fall short of this goal, notably in preparing graduate doctors in their role as first-responders for managing patients with acute illness [2]. In this letter, we propose strategies to help the intensive care medicine (ICM) community address this training shortfall.

Regional variability in the provision and organisation of undergraduate intensive care (UGIC) teaching, previously reported, undermines educational consistency and effectiveness [3]. Findings from our recent mixed-methods study highlight this ongoing issue. Following ethics approval, we surveyed ICM educators in all intensive care units (ICUs) with student placements (n = 16) and conducted semi-structured interviews with medical school representatives (n = 6) throughout Ireland between December 2013 and February 2014.

Although ICM was mandatory in five medical schools, most placements (13 of 16 ICUs; 81.3 %) lasted 5 days or less. Five ICUs (31.3 %) lacked a formal student learning curriculum, and procedural training was largely absent in all units. There was minimal provision for either student feedback or teaching evaluation, and 15 units (93.8 %) had no formal process for student assessment. Insufficient staffing, funding, and teaching time were the most commonly reported barriers to student teaching.

The ICU is a unique learning environment, offering students clinical diversity and workplace experiences found infrequently elsewhere, while achieving measurable cognitive and procedural learning outcomes [4]. Notwithstanding this, our study of Irish UGIC education (E. O’Connor, 2013, unpublished data), echoing international reports, suggests that its value is not being fully harnessed. How might we improve current practice, thereby preparing students for managing acute illness?

First, key educational principles underpinning educational design should be applied to UGIC placements. These include clear articulation of expected learning outcomes, a learning curriculum, a description of teaching methods (ideally a combination of bedside, classroom, and eLearning activities), formal evaluation of teaching, and summative student assessment aligned to the expected learning outcomes [5].

Second, clear endorsement by medical schools of the value of UGIC teaching, mainly by providing protected time and resources for teachers, and ensuring appropriate placement duration, should be forthcoming. Our research suggests that the level of institutional support is still a major barrier to effective student teaching.

Finally, we can promote effective and efficient UGIC learning through educational research. This can be either quantitative, using educational interventions and objective measureable outcomes to ask “do students learn in the ICU?”, or qualitative research, exploring students’ learning experiences, asking questions such as “how do students learn in the ICU?”.

## Abbreviations

ICM, intensive care medicine; ICU, intensive care unit; UGIC, undergraduate intensive care
